# Case Report: Long lasting response with TKI for combined hepatocellular-cholangiocarcinoma

**DOI:** 10.3389/fonc.2025.1459705

**Published:** 2025-03-18

**Authors:** Chiara Deiana, Andrea Palloni, Mirta Mosca, Francesco Vasuri, Stefano Chillotti, Simona Tavolari, Dario De Biase, Giorgio Frega, Elisa Giovannetti, Giovanni Brandi

**Affiliations:** ^1^ Medical Oncology, Istituto di Ricovero e Cura a Carattere Scientifico (IRCCS) Azienda Ospedaliero-Universitaria di Bologna, Bologna, Italy; ^2^ Department of Medical and Surgical Sciences, University of Bologna, Bologna, Italy; ^3^ Dipartimento di Oncologia Medica, Azienda Socio Sanitaria Territoriale (ASST) Sette Laghi, Varese, Italy; ^4^ Pathology Unit, Istituto di Ricovero e Cura a Carattere Scientifico (IRCCS) Azienda Ospedaliero-Universitaria di Bologna, Bologna, Italy; ^5^ Solid Tumor Molecular Pathology Laboratory, Istituto di Ricovero e Cura a Carattere Scientifico (IRCCS) Azienda Ospedaliero-Universitaria di Bologna, Bologna, Italy; ^6^ Department of Pharmacy and Biotechnology (FaBit), University of Bologna, Bologna, Italy; ^7^ Osteoncology, Soft Tissue and Bone Sarcomas, Innovative Therapy Unit, Istituto di Ricovero e Cura a Carattere Scientifico (IRCCS) Istituto Ortopedico Rizzoli, Bologna, Italy; ^8^ Department of Medical Oncology, Cancer Center Amsterdam, Amsterdam UMC, VU University Medical Center (VUmc), Amsterdam, Netherlands; ^9^ Cancer Pharmacology Lab, Fondazione Pisana per la Scienza, Pisa, Italy

**Keywords:** combined hepatocellular-cholangiocarcinoma, TKI, immunotherapy, predictive tools, case report

## Abstract

Combined hepatocellular-cholangiocarcinoma (cHCC-CCA) is a rare primary liver cancer, with intermediate biological characteristics between hepatocellular carcinoma (HCC) and cholangiocarcinoma (CCA). Given its rarity and the lack of robust data from randomized clinical trials, treatment is not standardized, and the choice on how to best manage the disease is left to the expertise of each institution. In the metastatic setting, given the more aggressive behavior of the CCA component, the usual approach is to start treatment with chemotherapy instead of tyrosine-kinase inhibitors (TKIs). We present a case report on a Caucasian male with a poor response to first-line treatment with chemotherapy directed against CCA, but with an excellent and long overall survival (OS) of 71 months, thanks to HCC-directed treatment with TKI. Here, we highlight the difficulty in selecting an appropriate treatment upfront for this rare cancer and we also discuss future perspectives regarding predictive tools, especially considering the recent genomic analysis of cHCC-CCA, and regarding the potential use of immunotherapy and target therapy.

## 1 Introduction

Combined hepatocellular-cholangiocarcinoma (cHCC-CCA) is a rare entity, comprising 2 - 5% of primary liver cancers and displaying architectural and morphological patterns present both in hepatocellular carcinoma (HCC) and intrahepatic cholangiocarcinoma (iCCA) (1). The biological behavior of this neoplasm is often described as intermediate between the more aggressive iCCA and the more indolent HCC, with median overall survival (OS) ranging from 7.9 months to 20.5 months (2, 3). Given the rarity of this tumor and the lack of randomized clinical trials, treatment for any stage is not standardized, and a personalized approach is often preferred, with a general preference for targeting the CCA component at first, as it is often considered more aggressive than the HCC component. In the present paper we present the case report of a Caucasian male with a diagnosis of cHCC-CCA who was first treated with chemotherapy with very poor results and whose subsequent treatment with a tyrosine-kinase inhibitor (TKI) was associated with long lasting disease control and an OS of 71 months. Starting from this case report we then discuss in depth the possible treatment options and we underline the many open issues that arise when dealing with this rare entity.

## 2 Lesson from a clinical case: a long-lasting response with TKI

In 2017, a 68-year-old Caucasian male with a previous history of eradicated HCV infection presented at the Emergency Room with abdominal pain. No other comorbidities, surgical interventions or relevant family history were reported. A computed tomography (CT) scan revealed the presence of an 11 cm mass in the liver and, given the lack of metastatic spread and the presence of pain, the patient was directed to surgery, without prior biopsy. A right hepatectomy, wedge resection of 4^th^ hepatic segment and cholecystectomy were performed, and in the following days transient posthepatectomy liver failure was observed (grade A according to ISGLS).

At histopathological analysis, two nodules were found in the liver: a small 2.2 cm cHCC-CCA nodule (acc. to WHO 2010) in the 4^th^ hepatic segment and a bigger 11 cm HCC nodule in the right liver, G3 (acc. to Edmondson). The cHCC-CCA nodule was defined as having two components: hepatocellular carcinoma G3 (acc. to Edmondson) with infiltrative growth and microtrabecular architecture and cholangiocarcinoma G2, and aspects of stem cell intermediate subtype were observed. Negative surgical margins were observed for both nodules, but unfortunately no lymphoadenectomy was performed, so nodal involvement at the time of diagnosis was unknown.

At the time, there was no standardized adjuvant treatment for CCA, thus given the good general condition of the patient and the post-hepatectomy liver failure, adjuvant treatment with one-year adjuvant metronomic capecitabine (500 mg twice daily) was started, with only one adverse effect, a decreased platelet count G2 (according to Common Terminology Criteria for Adverse Events CTCAEs v. 5.0). Unfortunately, after seven months from the start of adjuvant treatment, the patient relapsed, with a CT scan showing the appearance of bilateral nodules in the lungs and an 8 mm nodule in the hepatic dome of the 4^th^ hepatic segment. On suspicion of oncologic relapse due to iCCA, usually the more aggressive component of the tumor, first-line treatment with Gemcitabine and metronomic Capecitabine was selected. The more standard option of combining Gemcitabine plus a platinum derivate was forgone due to expected hematological toxicity of the doublet, however the use of gemcitabine monotherapy was deemed insufficient given the patients good performance status and adequate liver function, thus the combination of Capecitabine (500 mg twice daily) with Gemcitabine (1000 mg/m2 D1-8-15, Q28) was attempted.

Treatment was not well tolerated due to hematological toxicity, requiring numerous delays in chemotherapy administration and dose reduction (70%), which may have impaired efficacy of the first-line treatment. Furthermore, after the first CT scan after the start of 1^st^ line treatment revealed an increase in the number and diameter of the pulmonary nodules, although the nodule in the liver was no longer visible (**Table 1**). Time to disease progression on Gemcitabine - metronomic Capecitabine was 3.5 months.

**Table 1:** Evolution of secondary lesions during treatment.

	29/03/18	08/08/18	14/11/18	14/03/19	22/05/19	30/08/19	23/01/20	18/06/20	21/10/20	02/04/21	04/08/21	20/10/21	23/02/22	15/06/22	26/08/22	11/11/22
**TL1**	19x13	25x16	22x12	24x14	25x18	21x13	22x12	22x12	21x19	24x17	44x24	45x26	41x28	51x28	Not evaluable due to pleural effusion	Not evaluable due to pleural effusion
**TL2**	9x8	17x12	22x17	30x17	32x20	38x19	40x23	40x25	41x18	44x24	46x24	48x27	78x70	80x76	94x78	120x90
**TL3**							21x11	21x11	18x7	22x7	27x8	28x10	38x26	41x28	41x26	45x34
**Nodal TL1**	26x19	40x22	46x28	41x26	38x30	34x22	40x22	40x22	40x16	42x16	46x18	46x16	48x21	54x20	54x24	54x27
**Nodal TL2**		26x31	21x19	19x15	19x15	15x12	10x10									
**Nodal TL3**		25x21	23x21	23x18	23x19		28x24	28x24	30x25	32x26	33x30	35x31	32x25	32x25	34x28	35x30
**HL1**	12															
**Notes**							New lesions					New lesions	New lesions		Global increase of all non-target lesions	Carcinomatous lymphangitis
**RECIST 1.1**	**PD**	**PD**	**SD**	**SD**	**SD**	**SD**	**PD**	**SD**	**SD**	**SD**	**PD**	**PD**	**PD**	**SD**	**PD**	**PD**
	First evaluation of metastatic disease (during adjuvant treatment with metronomic Capecitabine).															
	Evaluation during treatment with Gemcitabine + metronomic Capecitabine.															
	Evaluation during treatment with Sorafenib.															
	Evaluation during treatment with Regorafenib.															
	Evaluation during treatment with Cabozantinib.															
	Evaluation during treatment with Cabozantinib + metronomic Capecitabine.															
	Evaluation during treatment with Metronomic Capecitabine.															

All lesions are expressed in millimeter (mm). Dates are expressed as DD/MM/YY.

Adapted from Recist criteria vs 1.1 (3 lesions were allowed per organ instead of just 2, due to the long history and evolution of the disease over time).

TL1: pulmonary target lesion 1, medial-posterior basal segment of the right lower lobe.

TL2: pulmonary target lesion 2, lateral basal segment of the right lower lobe.

TL3: pulmonary target lesion 2, lower left lobe.

Nodal TL1: nodal target lesion, subcarinal lymph nodes.

Nodal TL2: nodal target lesion 2, left hilar lymph nodes.

Nodal TL3: nodal target lesion 3, Lymph nodes of the Barety’s space.

HL1: hepatic target lesion 1, on the hepatic dome of the 4^th^ segment.

Given the unsatisfactory results of chemotherapy directed toward the iCCA component, treatment targeting the HCC component with Sorafenib (600 mg daily) was initiated, with good tolerability except for decreased platelet count G2, leading to a decrease in dosage (400 mg daily). After 16.9 months of stable disease, new pulmonary nodules and pathological mediastinal lymph nodes were observed, and 3^rd^ line treatment with Regorafenib (120 mg D1-21, Q28) was initiated. Once more, during TKI treatment, the disease remained stable for 18.1 months.

Due to pulmonary progression, a 4^th^ line of treatment with Cabozantinib (60 mg daily) was administered but with poor results, as after 3.6 months the disease had further progressed. Given the previous hematological toxicity, treatment with Cabozantinib was strengthened by the addition of metronomic Capecitabine (500 mg twice daily), obtaining with the doublet a progression free survival of 5.9 months.

**Figure 1:**
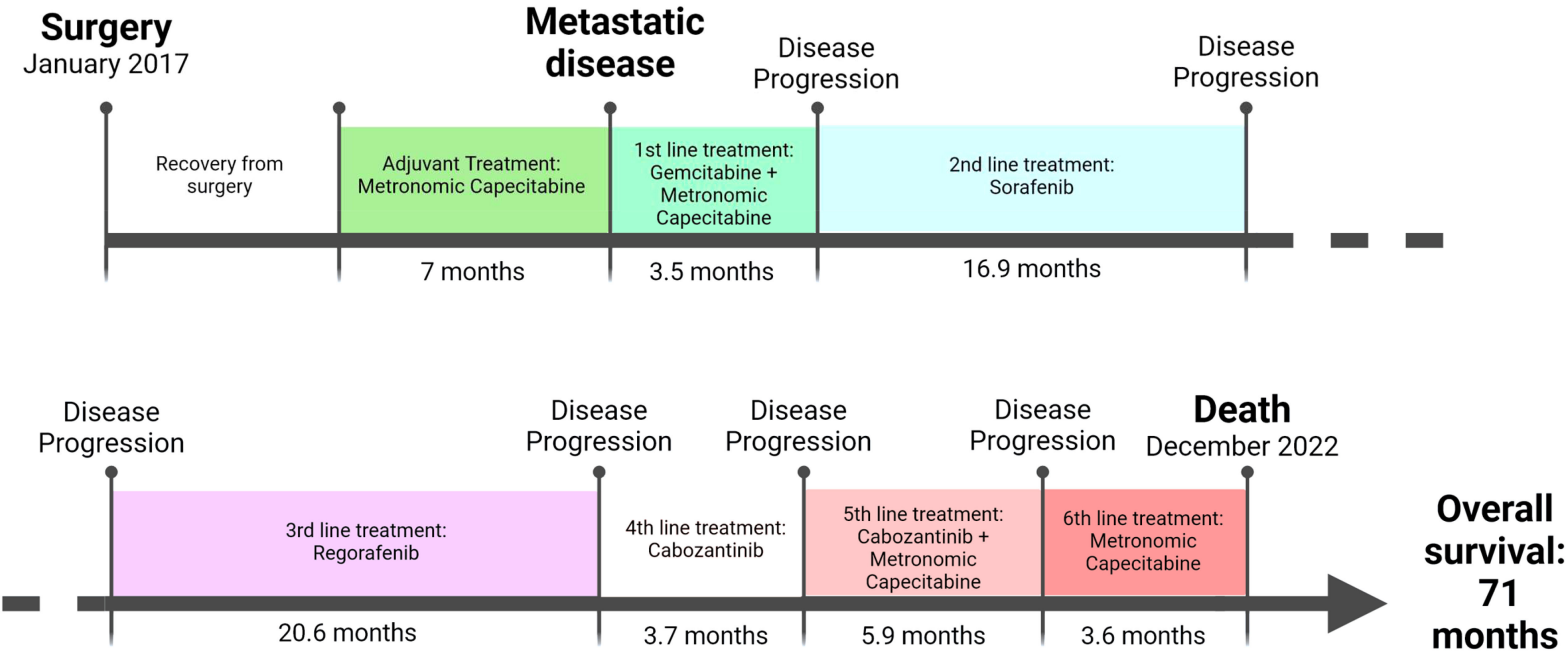
Timeline of active treatments administrated to the cHCC-CCA patient.

Due to further pulmonary progression, in the hopes of finding a targetable molecular alteration, next generation sequencing (NGS) was performed on the original pathological specimens. As reported in **Table 2**, C228T mutation in the promoter region of TERT gene was found both in cHCC-CCA and HCC nodules, possibly suggesting a common origin of both nodules. Furthermore, in the HCC nodule, CTNNB1 p.Lys335Thr and TP53 p.Gln100ArgfsTer23 mutations were also detected. Unfortunately, none of these mutations were susceptible for a target drug. A request for off label immunotherapy was therefore forwarded to the Italian regulatory agency, but it was ultimately denied. Treatment with metronomic Capecitabine was continued due to clinical benefit and good tolerability, while Cabozantinib was discontinued. In the following months the patient’s condition gradually worsened and the patient died in December 2022; OS from diagnosis was almost 6 years (71 months, **Figure 1**).

**Table 2:** NGS results on original cHCC-CCA and HCC specimens.

	TERT	VAF	CTNNB1	VAF	TP53	VAF
**cHCC-CCA nodule**	C228T	27	WT	/	WT	/
**HCC nodule**	C228T	52	p.Lys335Thr	18	p.Gln100ArgfsTer23	33

VAF, Variant Allele Frequency.

Undoubtedly, the long survival of this patient is highly unusual for a cHCC-CCA case, and it highlights how heterogeneous this disease can be, especially regarding response to therapy. It is easy to hypothesize that all metastases arose from the HCC component, however this hypothesis is not necessarily true. For example, a nodule in the liver was observed, but then it disappeared after first-line treatment with Capecitabine and Gemcitabine, a clinical behavior more consistent with an iCCA metastasis, although also compatible to a HCC clone sensitive to chemotherapy (4). Furthermore, during the last months of the disease, a brief disease stability was obtained only after the addition of metronomic Capecitabine to the already ongoing treatment with Cabozantinib. Unfortunately, common biomarkers for CCA or HCC (CEA, CA 19.9, AFP) were not tested and a biopsy of the pulmonary metastases was deemed too invasive, thus the nature of the secondary lesions was never revealed, underlining the need to develop a non-invasive tool to monitor the nature of the secondary lesions.

## 3 Treatment scenarios for cHCC-CCA

In the 5^th^ edition of Digestive system tumors of the World Health Organization (WHO), cHCC-CCA is defined as a primary liver cancer with the unequivocal presence of both hepatocyte and cholangiocyte differentiation within the same tumor (5). It often presents as a hepatic mass, sometimes accompanied by systemic symptoms such as weight loss, abdominal pain, and obstructive jaundice (6). Radiological features are dependent on the more represented histological component (6). Alpha-fetoprotein (AFP) can be elevated in 62% of cases and CA 19-9 in 22% of cases, while the rise of both tumor markers is rarer, around 15% of cases (7). Risk factors for cHCC-CCA are similar to the more common HCC and iCCA, such as viral hepatitis, cirrhosis, obesity, non-alcoholic steatohepatitis (NASH) and alcohol abuse; in addition, this malignancy can sometimes occur after HCC treatment with trans-arterial chemoembolization (TACE).

In the last years, clinical characteristics and prognosis of cHCC-CCA, HCC and iCCA, have been widely analyzed, with most studies reporting cHCC-CCA having a worse prognosis than HCC and a better prognosis than iCCA (7, 8). Tang et al. conduced a large study on 1041 patients diagnosed with either cHCC-CCA (135 patients), HCC (698 patients) or iCCA (208 patients), almost all (98.3%) treated with surgical resection, with the intent to compare their clinical features and survival outcomes (3). The authors found that cHCC-CCA presented an intermediate prognosis between HCC and iCCA, with a 1-year OS rates of 63.9%, 86.7% and 47.2% for cHCC-CCA, HCC and iCCA, respectively. The median OS was 20.5 months for cHCC-CCA patients, 35.7 months for HCC and 11.6 months for iCCA. Lymph nodes infiltration and the lack of TACE were independent factors associated with worse prognosis in the cHCC-CCA group. Baseline features of cHCC-CCA patients were similar to HCC patients regarding age, gender, incidence of hypertension, diabetes, cirrhosis, while the incidence of hepatitis B and C in cHCC-CCA patients was lower than that of HCC, but similar to that of iCCA (3).

Regarding treatment in the localized setting, patients with preserved liver function, acceptable portal hypertension and sufficient potential liver remnant, are usually treated with surgical resection with dissection of loco-regional lymph nodes (9). Unfortunately, the 5-year survival rate is only 30%, with a recurrence rate of 78% (10) and there are no established data about neoadjuvant or adjuvant treatment, although several treatments have been tried, from locoregional therapies to systemic chemotherapy and target drugs (11).

Similarly to HCC cases, liver transplantation can be considered as an alternative to surgery in patients who meet the Milan criteria (single nodule less than 5 cm; less than 3 nodules, largest nodule < 3 cm) (12). Although the quality of data is poor, it seems to suggest comparable survival rates; for example, a paper on 76 cHCC-CC patients who underwent resection (68 pt) or liver transplant (8 pt) shows 5-year disease free survival rates of 26.2% vs 37.5% (p = 0.333) and 5-year OS of 42.1% vs 50% (p = 0.591) (13). Further supporting the idea of similar outcomes, a meta-analysis comparing cHCC-CC cases treated with surgery vs liver transplantation found no significant differences in either tumor recurrence rate (65% vs 54% respectively) or 5 years OS (29% vs 41% respectively) (14).

Locally advanced or recurrent cHCC-CCA can also potentially be considered for loco-regional therapies such as image-guided ablation, TACE, radioembolization and hepatic arterial infusional chemotherapy. Data on the use of these treatments for cHCC-CCA is scarce and the rationale for their use is mostly being extrapolated from their use in HCC or iCCA cases (15). In a retrospective study, Mukund et al. (16) compared data from 13 patients with inoperable cHCC-CCA (due to tumor size, comorbidities or patient’s preference), 15 with iCCA and 101 with HCC, all treated with the same locoregional therapies (TACE, microwave ablation and TACE with radiofrequency ablation). Using propensity score matching, they recorded a shorter PFS for patients with cHCC-CCA vs patients with HCC (1.5 months versus 7.5 months), and shorter PFS for patients with iCCA than for patients with HCC (6 months versus 14 months). Similar results were reported for OS (12 months in cHCC-CCA and 28 months in HCC, 18 months in iCCA and 34 months in HCC) and for objective response.

More information is available on TACE specifically, with one retrospective study (17) on relapsed patients, showing survival outcomes poorer than the HCC control cases, while in another trial on inoperable cHCC-CCA (18), 70% of patients obtained a reduction or stability of the malignancy and an OS of 12.3 months; in both trials better results were seen in tumors with high vascularity.

In the metastatic setting, no standard of care exists and available data are extrapolated from small retrospective trials and case reports. The drugs most commonly used are those given in CCA cases, like Cisplatin with Gemcitabine or with Fluorouracil, or drugs more typically used in HCC, like Sorafenib. However, other regimens have been reported in literature, including Bevacizumab, Mitoxantrone, Epirubicin, S-1 (19). One of the largest studies is by Trikalinos et al., including 68 patients with metastatic cHCC-CCA (20). Sixteen patients received Gemcitabine alone or in combination with 5-Fluorouracil, 41 received Gemcitabine with a platinum derivate, 7 received Sorafenib and 4 were treated with other drugs. Median OS was 11.7 months in Gemcitabine + 5-Fluorouracil group, 11.5 months in Gemcitabine - platinum group and 9.6 months in Sorafenib group. Interestingly, disease control rate (DCR) was higher in the Gemcitabine-platinum group (78.4%) compared to Fluorouracil group (38.5%), while Sorafenib group obtained only 20% in DCR (although only 5 patients were evaluable for response) (20).

The superiority of platinum-based regimens over Sorafenib has also been reported in a retrospective trial of 36 advanced cHCC-CCA (21). Twelve patients treated with Cisplatin plus Gemcitabine obtained an OS of 10.2 months, 11 patients treated with Cisplatin- 5 Fluorouracil reached an OS of 11.9 months, and 5 patients treated with Sorafenib monotherapy had an OS of 8.1 months. Overall, treatment with Sorafenib was associated with poorer outcomes compared to those patients receiving a platinum-based chemotherapy regimen (hazard ratio: 4.49, 95% CI: 1.07-18.92; *P* = 0.041). However, in another larger retrospective European registry, the advantage of using a first-line chemotherapy versus non chemotherapy treatment was only seen as a trend toward better OS (15.5 vs 5.3 months, p = 0.052) and it was not confirmed at multivariate analysis when comparing chemotherapy versus Sorafenib, again indicating the uncertainty in first-line treatment for cHCC-CCA (22).

In the light of the recent approval of immunotherapy in the setting of both CCA and HCC (23–25), a new interest has risen to define whether cHCC-CCA may benefit from these drugs as well. An initial step into answering this question was conducted by a multicentric study on 96 cHCC-CCA samples (25). By gene expression profiling, two immune subtypes of cHCC-CCA were described: immune-low (43% of samples) and immune-high (57% of samples), with the latter being characterized by the upregulation of genes related to the adaptive and innate immunity, antigen presentation, immune suppression and inflammation. At multivariate analysis, the immune-high subgroup was associated with better outcomes in resected patients (HR = 0.17, 95% CI = 0.05–0.53, *P* = 0.002) (26). The authors hypothesized that this subgroup may benefit from immunotherapy but, given that this trial was not designed to assess response to actual treatment with immunotherapy, more trials should be carried out correlating the immune profile to the response to immunotherapy. A recent case report on 6 patients with cHCC-CCA treated with Atezolizumab + Bevacizumab showed promising results, with two patients obtaining a partial response and one patient having stable disease; however three patients had to discontinue treatment due to adverse events (27).

Given these initial results on immunotherapy and the data on better OS associated with platinum-based chemotherapy, it could be interesting to see if better survivals can be obtained with the standard first-line treatment for CCA, consisting of Cisplatin-Gemcitabine plus immunotherapy (23, 24).

Another important issue to consider is the genetic profile of these tumors, as the molecular landscape is heterogeneous, including loss of heterozygosity at 3p and 14q chromosomes, TP53 inactivation and TGF-β activation, mutation of KRAS, TERT promoter, WNT pathway, ARID1A and ARID2 (28). Recently, Murugesan et al. (29) analyzed the genomic profiles of cHCC-CCA (73 cases), iCCA (4975 cases) and HCC (1470 cases) and then used the data to create a machine learning model to classify cHCC-CCA as HCC-like or iCCA-like. Using this model, 58% of cHCC-CCA were classified as HCC-like, 16% as iCCA-like and 26% as ambiguous. These distinctions could explain why some patients respond better than others to iCCA therapies, while others benefit more from HCC therapies. Furthermore, according to this study, up to 24.6% of cases had some targetable alteration (BRCA2, ERBB2, IDH1, BRAF, FGFR2, and MET) (29). Of note, if these findings will be confirmed in further studies, they could pave the way for a new management of this disease, introducing NGS analysis as a standard tool in the decision-making paradigm for these patients.

Furthermore, in metastatic patients, whether or not the histology of the secondary sites resembles that of the primary lesions remains an open question. A case series on four patients revealed that phenotypes of metastases can vary, with some lesions maintaining a cHCC-CCA histology while others having a pure CCA or HCC component. The scenario can be even more complex, as a patient showed several metastases, some with HCC component and one having CCA differentiation (30). This example of heterogenous presentation highlights the difficulty in selecting the most appropriate systemic therapy, especially when histological analysis of the relapsed tumor or metastatic sites is not available.

## 4 Unmet clinical needs

To conclude, many open issues still need to be addressed by the scientific community regarding the treatment for cHCC-CCA. Firstly, the lack of a predictive tool to select which patients will benefit from therapy directed against the HCC versus the CCA component. Some initial steps have already been made, for example with the machine learning program by Marugesan et al. analyzing the genetic profile of cHCC-CCA; however, this tool still needs to be validated with clinical data on response to therapy and then with robust clinical trials to assess its effectiveness. Secondly, there is also a strong need to develop a non-invasive tool to monitor the nature of the metastatic disease, to better understand which component is more aggressive at each moment and whose treatment should be prioritized over the other.

Thirdly, the use of NGS to search for targetable mutations should be more standardized, and the use of target drugs implemented, as data on this subject are scarce due of the lack of clinical trials including cHCC-CCA patients. Furthermore, the use of NGS on all metastatic lesion to explore the clonality of the lesions could also help us understand the biological behavior of this rare cancer. Lastly, although treatment with immunotherapy was denied by the Italian regulatory agency for our patient, we believe that this type of treatment may deserve further investigations, given the good results on both HCC and CCA.

Overall, our case report highlights the many open questions that still remain in the treatment of cHCC-CCA, and the development of more predictive tools and effective treatments is mandatory for this rare disease to improve the outcome of these patients.

## Data Availability

The original contributions presented in the study are included in the article files, further inquiries can be directed to the corresponding author.
